# CuInS_2_ Quantum Dot and Polydimethylsiloxane Nanocomposites for All‐Optical Ultrasound and Photoacoustic Imaging

**DOI:** 10.1002/admi.202100518

**Published:** 2021-09-16

**Authors:** Semyon Bodian, Richard J. Colchester, Thomas J. Macdonald, Filip Ambroz, Martha Briceno de Gutierrez, Sunish J. Mathews, Yu Man Mandy Fong, Efthymios Maneas, Kathryn A. Welsby, Ross J. Gordon, Paul Collier, Edward Z. Zhang, Paul C. Beard, Ivan P. Parkin, Adrien E. Desjardins, Sacha Noimark

**Affiliations:** ^1^ Department of Medical Physics and Biomedical Engineering University College London London WC1E 6BT UK; ^2^ Wellcome/ESPRC Centre for Surgical and Interventional Sciences University College London Charles Bell House, 67–73 Riding House Street London W1W 7EJ UK; ^3^ Materials Chemistry Centre Department of Chemistry University College London 20 Gordon Street London WC1H 0AJ UK; ^4^ Department of Chemistry and Centre for Processable Electronics Imperial College London London W12 0BZ UK; ^5^ Johnson Matthey Technology Centre Sonning Common Reading RG4 9NH UK; ^6^ Central Laser Facility Harwell Science and Innovation Campus Chilton Didcot OX11 0DE UK

**Keywords:** multimodality imaging, nanocomposite coatings, optical fibers, quantum dots, ultrasound imaging

## Abstract

Dual‐modality imaging employing complementary modalities, such as all‐optical ultrasound and photoacoustic imaging, is emerging as a well‐suited technique for guiding minimally invasive surgical procedures. Quantum dots are a promising material for use in these dual‐modality imaging devices as they can provide wavelength‐selective optical absorption. The first quantum dot nanocomposite engineered for co‐registered laser‐generated ultrasound and photoacoustic imaging is presented. The nanocomposites developed, comprising CuInS_2_ quantum dots and medical‐grade polydimethylsiloxane (CIS‐PDMS), are applied onto the distal ends of miniature optical fibers. The films exhibit wavelength‐selective optical properties, with high optical absorption (> 90%) at 532 nm for ultrasound generation, and low optical absorption (< 5%) at near‐infrared wavelengths greater than 700 nm. Under pulsed laser irradiation, the CIS‐PDMS films generate ultrasound with pressures exceeding 3.5 MPa, with a corresponding bandwidth of 18 MHz. An ultrasound transducer is fabricated by pairing the coated optical fiber with a Fabry–Pérot (FP) fiber optic sensor. The wavelength‐selective nature of the film is exploited to enable co‐registered all‐optical ultrasound and photoacoustic imaging of an ink‐filled tube phantom. This work demonstrates the potential for quantum dots as wavelength‐selective absorbers for all‐optical ultrasound generation.

## Graphical abstract

Bilayer nanocomposites comprising CuInS_2_ quantum dots within micron‐scale polydimethylsiloxane host layers are microfabricated with dip‐coating on the distal ends of optical fibers. The wavelength‐selective optical absorption (high from 400 to 600 nm; low from 700 to 1200 nm) is well‐suited to multimodality optical ultrasound and photoacoustic imaging of biological tissue structures such as atherosclerotic plaque.

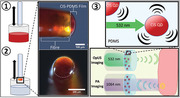

## 1 Introduction

Highly optically absorbing elastomeric nanocomposites can be formed as free‐standing materials, applied as thin films on macro^[^ ^1^ ^]^ and microscopic targets^[^ ^2^ ^]^ and patterned using surface modification techniques such as soft lithography.^[^ ^3^ ^]^ They are widely used in applications ranging from light‐emitting diodes^[^ ^4^ ^]^ to bio detection^[^ ^5^ ^]^ and solar cells.^[^ ^6^ ^]^ These elastomeric composites have shown great promise in biomedical imaging, in particular for optical ultrasound (OpUS) generation.^[^ ^7^, ^8^, ^9^, ^10^, ^11^ ^]^ Here, the elastomeric composite film absorbs a pulsed or modulated light source generating ultrasound waves via the photoacoustic effect.^[^ ^10^, ^12^, ^13^ ^]^ OpUS transmitters are advantageous since they can generate high ultrasound pressures and bandwidths from miniature devices without compromising their generation efficiencies; additionally, they are immune to electromagnetic interference and have the potential for low‐cost production.^[^ ^8^, ^14^, ^15^ ^]^ OpUS transmitters commonly take the form of a composite material using a polydimethylsiloxane (PDMS) host. PDMS is used due to its high volumetric thermal expansion coefficient which leads to efficient ultrasound generation. However, since it is optically transparent, optical absorbers must be integrated into PDMS to provide strong optical absorption for OpUS generation. For this purpose, many materials from metallic through to carbonaceous nanoparticles and organic dyes have been integrated into PDMS to fabricate nanocomposites for use as OpUS transmitters.^[^ ^13^, ^14^, ^16^, ^17^, ^18^, ^19^, ^20^ ^]^

Multiwall carbon nanotube‐polydimethylsiloxane (MWCNT‐PDMS) nanocomposites have been demonstrated as efficient OpUS transmitters which are attributed to their high optical absorbances^[^ ^21^ ^]^ and large thermal expansion coefficients.^[^ ^22^ ^]^ Previously, they have been coated onto a range of substrates, both macro‐ and microscopic, forming planar and fiber‐optic OpUS transmitters with applications spanning 3D biological imaging^[^ ^23^ ^]^ and high intensity focused ultrasound.^[^ ^24^, ^25^ ^]^ This has enabled high‐resolution imaging both ex vivo^[^ ^14^ ^]^ and in vivo^[^ ^15^ ^]^ using optical fiber devices. However, one limitation of MWCNT composites is their broad optical absorption profile which prevents optical transmission through the coatings for complementary secondary modalities, including imaging and therapeutic, such as optical spectroscopy or photodynamic therapy.

To address this and enable these types of multiple modalities within a single imaging device, engineering elastomeric nanocomposites with highly tuned and narrow optical absorption profiles is key.^[^ ^13^ ^]^ Light absorbed at a specific wavelength can be used to generate ultrasound while transmitted light is used for a complementary modality, such as photoacoustic (PA) imaging. These wavelength‐selective nanocomposites would enable a combination of various modalities, within a single miniature device, such as OpUS and PA imaging which could be used for diagnosing atherosclerotic plaque.^[^ ^9^, ^26^, ^27^ ^]^ Recently, it has been demonstrated that fiber‐optic OpUS transmitters composed of PDMS composite films containing crystal violet dye molecules and gold nanoparticles can be used to perform OpUS and PA ex vivo imaging of swine and human tissue.^[^ ^13^ ^]^ However, some limitations of these coatings were photobleaching and low ultrasound pressures, respectively.

Quantum dots (QDs) have promising properties for use in multimodality imaging including good photostability^[^ ^28^, ^29^ ^]^ and tuneable optical absorption profiles^[^ ^30^, ^31^ ^]^ allowing for high absorbance at wavelengths employed by lasers for OpUS generation. Their narrow absorption profiles can be tuned by altering the size and composition of the QDs.^[^ ^30^, ^32^, ^33^, ^34^, ^35^ ^]^ QDs can be functionalized for integration into PDMS^[^ ^36^ ^]^ and their photoluminescence quantum yields can be minimized through ligand engineering. The high quantum yields of QDs have long been exploited in optical applications such as photovoltaic cells,^[^ ^37^ ^]^ light‐emitting diodes,^[^ ^38^ ^]^ and photocatalysts.^[^ ^39^, ^40^ ^]^ However, for OpUS transmitters, QDs featuring low photoluminescence quantum yields are desired since the absorbed optical energy must be converted into heat via the PA effect to generate ultrasound rather than directed into photoluminescence pathways.^[^ ^41^ ^]^ Moreover, different synthetic routes can also influence their performance and they can be prepared by different approaches, for instance, hot‐injection,^[^ ^42^, ^43^ ^]^ room temperature‐injection,^[^ ^44^ ^]^ two‐step nucleation and growth,^[^ ^45^ ^]^ microwave,^[^ ^46^, ^47^ ^]^ ligand‐assisted reprecipitation,^[^ ^48^ ^]^ and the heat‐up method.^[^ ^49^ ^]^ However, the most suitable for large‐scale production remains the heat‐up method.^[^ ^49^, ^50^ ^]^ QDs with nonheavy metal elements such as CuInS_2_ (CIS) QDs are attractive alternatives to other popular QD species which feature heavy metal ions, in particular for biomedical applications^[^ ^32^, ^51^, ^52^ ^]^ since they do not contain toxic metals such as lead or cadmium.

In this paper, we present a novel QD nanocomposite for OpUS generation, the first of its kind for this application. The composite comprised CIS QDs incorporated into a PDMS host and exhibited a wavelength‐selective absorption. By coating the distal tip of optical fibers with the composite, OpUS generators were fabricated. These were used to demonstrate co‐registered OpUS imaging and PA imaging using an ink‐filled tube phantom.

## 2 Methodology

### 2.1 CIS QDs Synthesis

CIS QDs were synthesized using a method adapted from that detailed by Li et al.^[^ ^53^ ^]^ Briefly, copper iodide (CuI, 0.190 g, 1 mmol, Sigma Aldrich, UK), indium acetate (InAc_2_, 0.293 g, 1 mmol, Sigma Aldrich, UK), 1‐dodecanethiol (1‐DDT, 1 mL, 4 mmol, Sigma Aldrich, UK), and octadecene (ODE, 4 mL, Sigma Aldrich, UK) were reacted together inside a three‐necked round‐bottomed flask. The flask was degassed under a vacuum and then purged with nitrogen gas three times for 5 min to remove any unreacted gases. The reaction mixture was heated to 150 °C for 10 min, forming a clear yellow solution. Subsequently, the temperature was increased to 200 °C whereby the reaction mixture changed in color from clear yellow through orange to dark red. The reaction mixture was cooled to room temperature, preventing any further growth in the CIS QD cores. The QDs were washed with acetone (3:1, acetone: QD) and centrifuged (4500 rpm, 10 min), after which the supernatant was decanted. The washing process was repeated twice more, after which the QDs were resuspended in toluene.

### 2.2 Optical Fiber Coating

A CIS‐PDMS coating was created on the end face of optical fibers. PDMS (Med‐1000, Polymer Systems Technology, UK) was combined with toluene in a ratio of 0.5 g:0.9 mL, respectively. The mixture was thoroughly stirred until a homogenous and clear solution was formed. Optical fibers (200 µm core diameter, FG200LCC, Thorlabs, UK) were prepared by stripping the buffer coating from the distal end exposing the cladding. Subsequently, the fibers were cleaned using isopropanol (IPA) then cleaved. A CIS QD paste was created by evaporating off the toluene solvent in a fume hood under constant airflow. The optical fiber end face was dip‐coated with the CIS QD paste forming an oily coating. The fiber was subsequently overcoated by dip‐coating in the prepared PDMS solution, forming a CIS‐PDMS bilayer film. The CIS‐PDMS‐coated optical fibers were dried facing upward in ambient conditions for 24 h to allow the PDMS to cure.

### 2.3 Coating Examination

**Figure 1:**
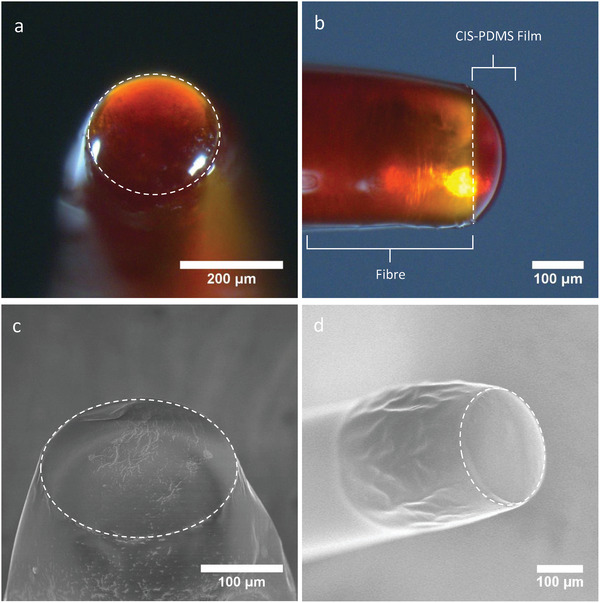
CIS‐PDMS fiber‐optic film fabricated by the dip‐coating method featuring a) end‐on and b) side‐on illumination. SEM images of c) end‐on and d) side‐on views of CIS‐PDMS film prepared with dip‐coating method.

All fiber‐optic coatings were examined using a stereomicroscope (Leica, UK). End‐on imaging of the films was used to study the homogeneity and surface topology (**Figure** **1a**). Both direct and through‐film illumination were used, giving an indication of film thickness and QD concentration. Side‐on imaging was used to analyze the coating morphology (Figure 1b). Additionally, a scanning electron microscope (SEM, JSM‐6301F, JEOL, JP) was used to obtain high‐resolution images of the CIS‐PDMS composite coatings (Figure 1c,d). Energy‐dispersive X‐ray spectroscopy (EDX) analysis was conducted using a transmission electron microscope (JEM 2800, JEOL, JP) operating at 200 kV with two EDX detectors (Johnson Matthey Technology Centre at Sonning Common).  EDX elemental data were obtained using Thermo Scientific X‐ray Microanalysis Software.

### 2.4 Optical Characterization

The optical absorbance of the CIS‐PDMS coatings was measured across a wavelength range of 400–1200 nm. Broadband light from a halogen lamp (HL‐2000‐HP‐FHSA, Ocean Optics, USA) was coupled into the coated optical fiber and the coated distal surface was inserted into an integrating sphere (FOIS‐1, Ocean Optics, USA). The output of the integrating sphere was measured with two spectrometers independently (Flame‐T‐VIS‐NIR and NIRQuest512, Ocean Optics, USA) to achieve a broader combined measurement bandwidth. A non‐coated cleaved fiber was used as a reference and dark measurements were taken to correct for background light.

### 2.5 Ultrasound Characterization

The ultrasound pressure generated by the CIS‐PDMS coatings upon illumination with a pulsed laser was measured at 532 and 1064 nm. For 532 nm, a Q‐switched Nd:YAG laser (FQ‐500‐532, Elforlight, UK) with a pulse length of 10 ns and pulse energy of 48.2 µJ (laser fluence at the coating: 153.4 mJ cm^−2^) was employed. For 1064 nm, a Q‐switched Nd:YAG laser (SPOT‐10‐500‐1064, Elforlight, UK) with a pulse length of 2 ns and a pulse energy of 20.8 µJ (corresponding laser fluence at the coating: 66.2 mJ cm^−2^) was coupled into the coated optical fiber. An ultrasound field scan was performed by scanning the hydrophone over a 2D grid orthogonal to the longitudinal axis of the optical fiber. The grid was measured 3 mm × 3 mm with an isotropic step size of 50 µm and was positioned at an axial distance of 1.5 mm from the coated distal tip of the optical fiber. The fiber under test was mounted opposite a calibrated needle hydrophone (diameter: 200 µm, Precision Acoustics Ltd., UK) with a calibration range of 1–30 MHz. The coated end face of the optical fiber was aligned with the hydrophone at a distance of 1.5 mm.

### 2.6 Dual‐Modality Imaging

#### 2.6.1 Phantom Preparation

A phantom was prepared by positioning three sections of polymethylpentene (PMP) tubing in a glass dish *≈*2.5 mm above the base of the dish (Figure S4b, Supporting Information). The petri dish was then placed on a hot‐plate heated to 160 °C. Molten gel‐wax was added to the dish until it reached the brim partially submerging a portion of the tubes (Figure S4b, Supporting Information). Subsequently, the phantom was left to cool to room temperature. Three aqueous solutions of Derussol carbon black (CB) with differing concentrations were prepared (concentrations: 0.1%, 0.0135%, 0.00151%). Each of the PMP tubes was filled with a different concentration solution and the ends of the tubes were sealed using a waterproof adhesive.

#### 2.6.2 Co‐Registered OpUS and PA Imaging

A fiber optic ultrasound transducer was fabricated by combining a CIS‐PDMS‐coated optical fiber with an FP fiber‐optic sensor.^[^ ^54^ ^]^ The fibers were held adjacent with their distal ends aligned. The fiber optic ultrasound transducer was mounted on a motorized translation stage with the distal end positioned above the tube phantom submerged in a water bath (Figure S5, Supporting Information). The FP sensor was interrogated using a system previously described.^[^ ^8^, ^13^, ^15^, ^55^ ^]^ Briefly, a continuous wave tuneable laser (wavelength: 1500–1600 nm, power: 5 mW, Tunics T100S CL, Yenista Optics, France) was coupled into the hydrophone via a circulator. Reflected signals were separated into low (<50 kHz) and high (>500 kHz) frequency components. The low‐frequency component was used to optimally bias the FP sensor, while the high‐frequency component was encoded with the ultrasound signals. For OpUS imaging, a Q‐switched Nd:YAG laser (wavelength: 532 nm, pulse length: 10 ns, repetition rate: 100 Hz, pulse energy: 39.5 µJ, Elforlight, UK) was coupled into the CIS‐PDMS‐coated optical fiber. For PA imaging, a Q‐switched Nd:YAG laser (wavelength: 1064 nm, pulse length: 2 ns, repetition rate: 100 Hz, pulse energy: 41.5 µJ, Elforlight, UK) was coupled into the CIS‐PDMS‐coated optical fiber.

To acquire co‐registered OpUS and PA images, a single line scan was repeated sequentially. For the first scan, the 532 nm laser was coupled into the CIS‐PDMS‐coated optical fiber, providing an OpUS image. Subsequently, the 1064 nm laser was coupled into the CIS‐PDMS‐coated optical fiber and the scan was repeated providing a PA image. The scan protocol was the same for both images, the transducer was translated across the phantom in 50 µm increments for 400 steps. At each step, an OpUS, or PA, A‐line was acquired.

The acquired data were processed and reconstructed into a co‐registered OpUS/PA image. For both OpUS and PA data, the acquired signals were frequency filtered (Butterworth, 4th order, bandpass: 2.5–25 MHz), followed by cross‐talk removal^[^ ^14^ ^]^ and time‐gain compensation. Cross‐talk arose due to the direct transmission of ultrasound from the transmitting fiber to the receiver fiber. This cross‐talk signal remained relatively constant for neighboring A‐lines, so could be removed using a general linear model, as with previous studies.^[^ ^14^, ^15^ ^]^ For PA images, an additional processing step was required due to residual ultrasound signal caused by absorption of the 1064 nm laser light within the CIS‐PDMS coating. The residual ultrasound was removed using a method similar to the cross‐talk removal. Here, the residual signal was modeled using the acquired OpUS image and then fitted to the PA image. The modeled signal was then subtracted leaving the PA signals. Following the signal processing, both OpUS and PA images were reconstructed using a k‐space method.^[^ ^14^, ^56^ ^]^ Finally, a composite image was constructed by displaying the PA image overlaid on the OpUS image.

The photostability of the coating was examined by measuring the cross‐talk signal voltage recorded by the FP sensor across the total A‐line scans taken during the gel‐wax phantom imaging.

## 3 Results

### 3.1 Coating Examination

All CIS‐PDMS coatings were domed in shape and an orange/red color characteristic of the CIS QDs (Figure 1a,b). End‐on fiber illumination indicates that the films were smooth with uniform coverage of the fiber tip. Using side‐on imaging, examination showed that coatings were *≈*50 µm thick at the thickest point of the PDMS dome (Figure 1b).

SEM images of the fiber tips covered with the CIS‐PDMS films displayed smooth surface morphologies (Figure 1c,d). In contrast, SEM images of fiber tips coated with solely the CIS QD underlayer displayed distinctive “bird‐feet” patterns which are thought to result from the fast evaporation of the residual toluene solvent from the CIS QD paste (Figure S1, Supporting Information). The highly viscous PDMS overcoat covered the patterns underneath providing the observed smooth film surface. EDX spectra of coating samples taken from the end‐face and sides of the fibers featured a range of high‐intensity peaks including those corresponding to copper (Cu), indium (In), and sulfur (S) species, characteristic of the presence of CIS QDs at the fiber end‐face and sides (Figure S3, Supporting Information).

### 3.2 Optical Absorption Spectra

**Figure 2:**
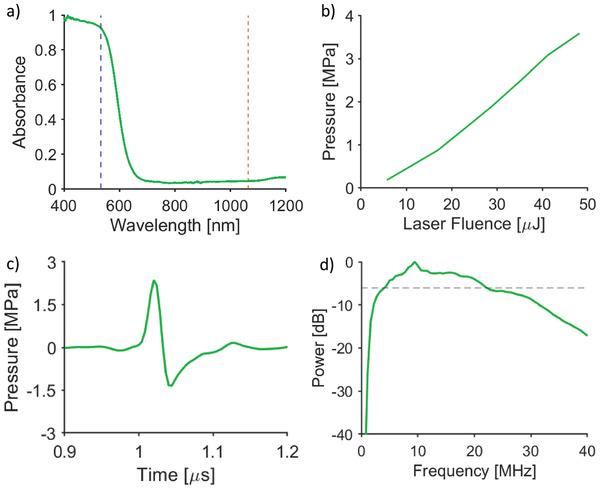
a) Absorption spectrum of CIS‐PDMS film produced by the dip‐coating method with absorption at 532 nm (blue dashed line) and 1064 nm (orange dashed line) highlighted. b) Ultrasound pressures measured from CIS‐PDMS coating with different laser pulse energies. c) Ultrasound wave plot and d) power spectrum of a CIS‐PDMS film produced by the dip‐coating method; the dashed line in (d) corresponds to –6 dB.

The optical absorption of all CIS‐PDMS coatings was measured. A wavelength‐selective optical absorption profile was observed characteristic of CIS QDs (**Figure** **2a**).^[^ ^57^, ^58^ ^]^ A high absorbance at 532 nm was followed by a steep absorption edge leading to lower absorbances at longer wavelengths such as 1064 nm. At 532 nm, the absorbance was *≈*90%, compared to *≈*5% at 700 nm, as measured across five samples. At wavelengths around 1064 nm, optical absorbance in the film was low (*A* = 5%), enabling the transmission of light for use in PA imaging.

### 3.3 Ultrasound Characterization

The ultrasound pressure generated by the CIS‐PDMS coatings was measured at a distance of 1.5 mm from the coating for increasing laser fluences. The peak‐to‐peak ultrasound pressure was found to increase linearly with increasing laser fluence and an ultrasound generation efficiency of 0.024 MPa mJ^−1^ cm^−2^ was found (Figure 2b). The maximum peak‐to‐peak ultrasound pressure when irradiated with a laser fluence of 153.4 mJ cm^−2^ was 3.7 MPa, with a corresponding −6 dB ultrasound bandwidth of 18 MHz (Figure 2c,d). Under illumination from greater laser fluences, the ultrasound pressure generated by the CIS‐PDMS coatings decreased, which was indicative of damage to the coatings. There was a low standard error in the pressure (0.25 MPa) generated across the test samples, demonstrating the consistency of the fabrication method. Further, the ultrasound field measurements showed that the generated ultrasound field was circularly symmetric (Figure S8, Supporting Information), with a full‐width half‐maximum beam width of ≈0.6 mm at a distance of 1.5 mm from the optical fiber. For excitation at 1064 nm, i.e., in the low optical absorption region, the generated ultrasound pressure amplitude was 25% of that measured at 532 nm. Ultrasound characterization results demonstrated that CIS‐PDMS coatings had good photostability under illumination at high laser fluences, with no decrease in generated ultrasound for incident laser pulse energies up to 48.2 µJ.

### 3.4 Dual‐Modality Imaging

OpUS and PA images were acquired over the central section of the tube phantom (Figure S5, Supporting Information). The surface of the gel‐wax was clearly defined in the OpUS image as a distinct boundary with the surrounding water (Figure S6b, Supporting Information). Further, the three tubes could be resolved. In the dual‐modality image, they each appeared as two axially narrow acoustic reflections, one from the top surface of the tube, and a second from the bottom surface. The edges of the tube were not resolved since only surfaces with a significant component perpendicular to the transmitted ultrasound directed reflections back toward the receiver.

**Figure 3:**
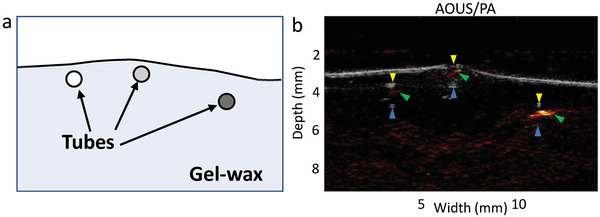
a) A diagram illustrating the positions of each of the three ink‐filled tubes within the gel‐wax medium. b) An Overlaid OpUS/PA image of the cross‐section of the phantom. Arrows indicate locations of signals arising from the tubes. US top tube boundary—yellow arrow, US bottom tube boundary—blue arrow, PA signal—green arrow.

The PA image was overlaid on the OpUS image to form an amalgamated image (**Figure** **3b**). PA signal was present for all three ink filled tubes, with signal intensity corresponding well to concentration in the original PA images (concentrations (v/v, %): signal‐to‐noise ratio (SNR) (dB); 0.00151%: 21 dB, 0.0135%: 25 dB, 0.1%: 35 dB). Further, the PA signals were well aligned with the locations of the tubes in the OpUS images. However, removal of the residual ultrasound signal led to a reduction in the SNR of the PA image and background noise and artifacts were present (Figure S6c, Supporting Information).

By comparison with the OpUS image (Figure S6b, Supporting Information), the corresponding PA image (Figure S6a, Supporting Information) contained a large, reflected ultrasound signal originating from the nonzero absorption of the 1064 nm laser light, by the CIS‐PDMS film, used to produce the PA signal. The processed PA image (Figure S6c, Supporting Information), where the ultrasound signal recorded in the OpUS image was subtracted from that recorded in the PA image, exhibited one bright spot which derived from one particular tube while two dimmer spots represented ultrasound emitted by the two other tubes featuring lower concentrations of the CB ink. Despite the removal of the background ultrasound signal, some noise remained which reduced the contrast of the PA signal from the phantom. When the OpUS image was overlaid with the corresponding PA image, all three tubes were more readily observed.

Both the peak positive and peak negative voltages, corresponding to the cross‐talk ultrasound signal recorded by the FP sensor, remained constant over the 400 A‐line scans performed indicating the steady generated ultrasound signal.

## 4 Discussion

This paper describes the fabrication and application of wavelength‐selective CIS‐PDMS QD nanocomposites applied to miniature optical fiber targets to demonstrate co‐registered OpUS and PA imaging. The bottom‐up fabrication method used results in full PDMS encapsulation of the CIS QDs dip‐coated onto the fiber. This is advantageous as the medical‐grade PDMS presents a smooth, biocompatible surface. This observation was supported by both the microscope and SEM images (Figure 1a–d) which show a smooth PDMS outer surface. Prior to use in a medical device, a more thorough investigation of the QD encapsulation should be carried out whereby the chemical stability and inertness of the coating are examined in operation over an extended timespan. Specifically, QDs encapsulated within films have been reported to leech ions from their structures which accumulate into the surrounding biological tissue and can then cause cell death.^[^ ^59^, ^60^, ^61^, ^62^ ^]^ Along the path to clinical translation, it would be pertinent to examine the degree of leaching of the CIS QDs from the PDMS matrix.

The fabricated CIS‐PDMS coatings exhibited narrow optical absorption profiles with a peak centered at 532 nm. For longer optical wavelengths (1064 nm), the optical absorption was low. This wavelength‐selective nature makes the composite well‐suited for use in multimodality imaging where a strong optical absorption at one wavelength can be used for ultrasound generation and low optical absorption at other wavelengths is necessary for the transmission of light for complementary modalities such as light‐activated therapy.^[^ ^13^ ^]^ Crucially, the absorption peak exhibited by the fabricated coatings correlates with a common excitation wavelength for OpUS at 532 nm. This wavelength is widely used due to the availability of short‐pulsed lasers at this wavelength.^[^ ^63^ ^]^ Additionally, the low optical absorption at longer wavelengths is well‐suited to the incorporation of many complementary modalities, including PA imaging with molecular optical contrast (blood: 600–900 nm, lipids: 1210 nm^[^ ^63^, ^64^, ^65^, ^66^ ^]^), near‐infrared spectroscopy for tissue characterization (600–1000 nm^[^ ^67^, ^68^ ^]^), and optical ablation for therapeutics (532–1064 nm^[^ ^66^ ^]^). This opens up avenues for the development of miniaturized functionalized devices for in vivo clinical imaging applications such as multimodality real‐time image guidance of surgical instruments during minimally invasive interventions.

The CIS‐PDMS coatings yielded clinically useful ultrasound (>3 MPa, >15 MHz). The high ultrasound pressures provided large imaging depths and were comparable to those produced by many other ultrasound fiber‐optic transmitters featured in previous studies, which have performed both high‐resolution in vivo and ex vivo imaging of tissue.^[^ ^8^, ^13^, ^14^, ^16^, ^20^ ^]^ To compare the generation efficiency with previous results, the 2D pressure field measurements were numerically backpropagated to the coating surface to yield a transduction efficiency of 0.024 MPa mJ^−1^ cm^−2^. While this efficiency was lower than those recently presented for carbon nanotube‐based coatings^[^ ^8^, ^16^ ^]^ (0.13–0.65 MPa mJ^−1^ cm^−2^) and graphene‐based coatings^[^ ^69^ ^]^ (0.21 MPa mJ^−1^ cm^−2^), the high damage threshold of the CIS‐PDMS coating allowed for comparable absolute pressures to be achieved. The generated pressure increased linearly with laser fluence, as expected from PA excitation.^[^ ^70^ ^]^ No decrease in generated ultrasound pressure associated with coating damage was observed in this study as demonstrated by the stable cross‐talk voltages recorded over the 400 A‐line scans taken during the joint OpUS/PA imaging of the gel wax phantom (Figure S7, Supporting Information). This combined with the long coating exposure times suggested good photostability for the QD materials. In addition to high pressure, the wide ultrasound bandwidths led to high spatial imaging resolutions as seen in the OpUS images. However, these bandwidths are narrower than demonstrated with some previously reported carbonaceous coatings.^[^ ^13^, ^14^, ^20^ ^]^ One possible reason for this could be the relatively large coating thickness, predominantly owing to the thick PDMS overcoat. The generated ultrasound bandwidth is proportional to the coating thickness and so the bandwidth might be improved in future studies by fabricating thinner coatings. This could be facilitated by lowering the coating solution viscosity. However, there is a trade‐off with optical absorption, which will decrease as the coating thickness is reduced. While this reduced absorption will lead to a decrease in the residual ultrasound signal in the PA images, it will also cause a reduction in the generated ultrasound pressure at 532 nm. To compensate for this reduced absorption, the laser pulse energy could be increased. However, transmitted light at 532 nm may lead to a confounding PA signal at this wavelength. This trade‐off could be explored in future work. Alternatively, the reduction in absorption might be mitigated in part by increasing the CIS QD concentration of the coating. Otherwise, the CIS‐PDMS composite may be well suited to applications where a narrower bandwidth or lower frequencies are preferred, such as neuronal stimulation,^[^ ^71^ ^]^ or in the context of pulse‐echo all‐optical ultrasound imaging in cases where lower bandwidth detectors are used.^[^ ^72^, ^73^ ^]^

The imaging performance of the CIS‐PDMS film fabricated by the dip‐coating method was examined by performing dual‐modality (OpUS and PA) imaging. In the co‐registered OpUS and PA image, the three tubes could be discerned (Figure 3b). The high ultrasound pressures led to OpUS images with high SNR enabling all three ink‐filled tubes to be resolvable. The PA image, however, had a lower SNR, which was partially due to the low laser power for PA excitation, but also from the need for extra processing to remove residual ultrasound signal generated in the coating at 1064 nm. This could be mitigated by reducing the optical absorption of the CIS‐PDMS coating at longer wavelengths near 1064 nm. Reducing the concentration of the CIS QDs integrated into the PDMS nanocomposite would provide a method of lowering the overall optical absorption of the CIS‐PDMS coating including that at 1064 nm. This may adversely affect the generated ultrasound pressures at 532 nm, however, given the magnitude of the ultrasound pressures produced with these OpUS fiber‐optic probes, a slight decrease in the pressures should not be overly detrimental to their clinical imaging capabilities. Other solutions include synthesizing CIS QDs with a narrower particle‐size distribution lowering the optical absorption at longer wavelengths (>532 nm). The amplitude of the residual ultrasound signal generated at 1064 nm was found to be 75% lower than that generated for the same laser fluence at 532 nm. This is higher than expected based on the measured optical absorption values alone. However, in a recent modeling study, Rajagopal and Cox demonstrated that for longer laser pulse durations, the generated ultrasound amplitude is decreased.^[^ ^74^ ^]^ In that sense, there is qualitative consistency with the difference in laser pulse durations used here (1064 nm: 2 ns duration; 532 nm: 10 ns). The residual ultrasound signal might be reduced further in future studies by using a laser with a shorter pulse length for photoacoustic signal generation.

The overlay image combining the OpUS and PA images demonstrates the potential for a greater range of information of the imaged tissue to be discerned. For example, this dual‐modality technique could be used in surgery to differentiate separate anatomical features such as blood vasculature and nerves.^[^ ^63^, ^75^, ^76^, ^77^, ^78^ ^]^ A major limitation of the imaging detailed here was that the OpUS and PA imaging were carried out sequentially. However, such a set‐up would not be possible when performing in vivo imaging. The movement of the target tissue would lead to a severely reduced image quality. This could be improved by interleaving the A‐line acquisition of the OpUS and PA modalities. This method would require additional optical equipment in order to couple both lasers into the same optical fiber. Alternatively, M‐mode imaging could be employed where a series of A‐line scans are concatenated to form an M‐mode image. An increased laser pulse repetition rate would be required to increase the image acquisition rate, this might be limited by heating within the coating. However, in previous PDMS composite studies, high repetition rates have been used without damage.^[^ ^16^, ^79^ ^]^ The results presented here provide a proof‐of‐concept for dual‐modality OpUS/PA imaging that sets the stage for in vivo application. The current work used motorized scanning to acquire two co‐registered images. For in vivo application with a minimally invasive procedure, lateral space constraints can be of critical importance and therefore it could be beneficial to deliver excitation light at different wavelengths sequentially, for instance using a fiber optic coupler or switch. By pairing this excitation light delivery fiber with a fiber‐optic ultrasound detector, multimodality sensing and imaging could be performed from needles, as used in previous OpUS studies,^[^ ^15^ ^]^ or alternatively, a flexible catheter.

## 5 Conclusion

Ultrasound was produced from an optical fiber whose distal end was coated with a bilayer CIS‐PDMS film deployed via a dip‐coating method. The coatings exhibited a strongly wavelength‐selective nature with high optical absorption at 532 nm for ultrasound generation and low optical absorption for wavelengths >1064 nm. Such a composite film is the first to include QDs of any type, for use in ultrasound generation. Using a 48.2 µJ, 2 ns, 532 nm laser pulse, ultrasound pressures of over 3 MPa and bandwidths breaching 15 MHz have been recorded, 1.5 mm from the film's surface. Such measurements compare favorably against other ultrasound transmitters that have performed both ex vivo and in vivo tissue imaging.^[^ ^13^, ^14^, ^15^ ^]^ The wavelength‐selective properties of the film enable co‐registered OpUS and PA imaging and will allow for the delivery of light to tissue for other modalities for diagnosis and therapy, with such targeted clinical applications such as the imaging and diagnosis of atherosclerotic plaque as well as the use of photodynamic therapy which has shown great promise as a cancer treatment.^[^ ^13^, ^80^ ^]^

## Conflict of Interest

The authors declare no conflict of interest.

## Supporting information

Supporting Information

## Data Availability

The data that support the findings of this study are available from the corresponding author upon reasonable request.
