# Supplementary material for: CuInS2 Quantum Dot and Polydimethylsiloxane Nanocomposites for All‐Optical Ultrasound and Photoacoustic Imaging
Source: Adv Mater Interfaces. 2021 Sep 16;8(20):2100518. doi: 10.1002/admi.202100518 (PMC8573612; doi:10.1002/admi.202100518)
Supplement: Supplementary file 1 — Supporting Information [file ADMI-8-2100518-s001.pdf]

© 2021 Wiley-VCH GmbH

ADVANCED MATERIALS INTERFACES

Supporting Information

for *Adv. Mater. Interfaces,* DOI: 10.1002/admi.202100518

$CuInS_2$ Quantum Dot and Polydimethylsiloxane Nanocomposites for All-Optical Ultrasound and Photoacoustic Imaging

Semyon Bodian, Richard J. Colchester,* Thomas J. Macdonald, Filip Ambroz, Martha Briceno de Gutierrez, Sunish J. Mathews, Yu Man Mandy Fong, Efthymios Maneas, Kathryn A. Welsby, Ross J. Gordon, Paul Collier, Edward Z. Zhang, Paul C. Beard, Ivan P. Parkin, Adrien E. Desjardins, and Sacha Noimark*

Supporting Information:

$CuInS_2$ Quantum Dot and Polydimethylsiloxane Nanocomposites for All-Optical Ultrasound and Photoacoustic Imaging

Semyon Bodian*, Richard J. Colchester*, Thomas J. Macdonald, Filip Ambroz, Martha Briceno de Gutierrez, Sunish J. Mathews, Yu Man Mandy Fong, Efthymios Maneas, Kathryn A. Welsby, Ross J. Gordon, Paul Collier, Edward Z. Zhang, Paul C. Beard, Ivan P. Parkin, Adrien E. Desjardins, Sacha Noimark

1. SEM Characterisation of $CuInS_2$ Coatings

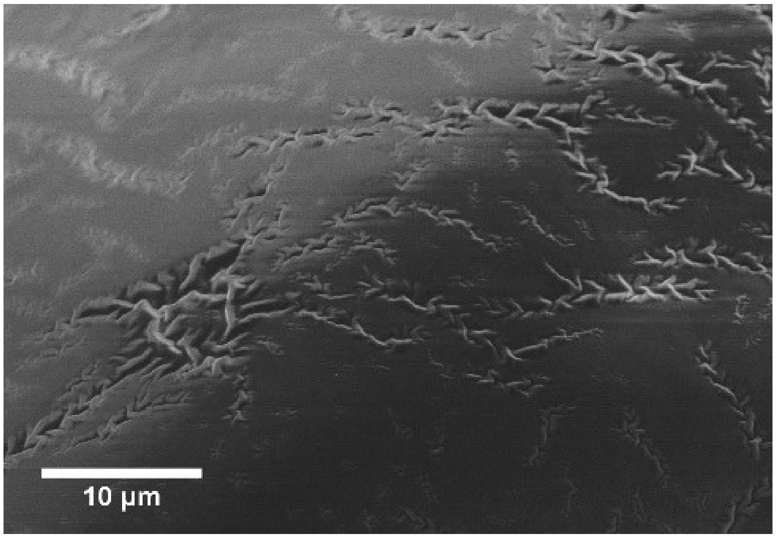

Figure 1): SEM image of fibre distal end coated with CIS QDs displaying “bird-feet” pattern.

2. TEM Characterisation of $CuInS_2$ Quantum Dots

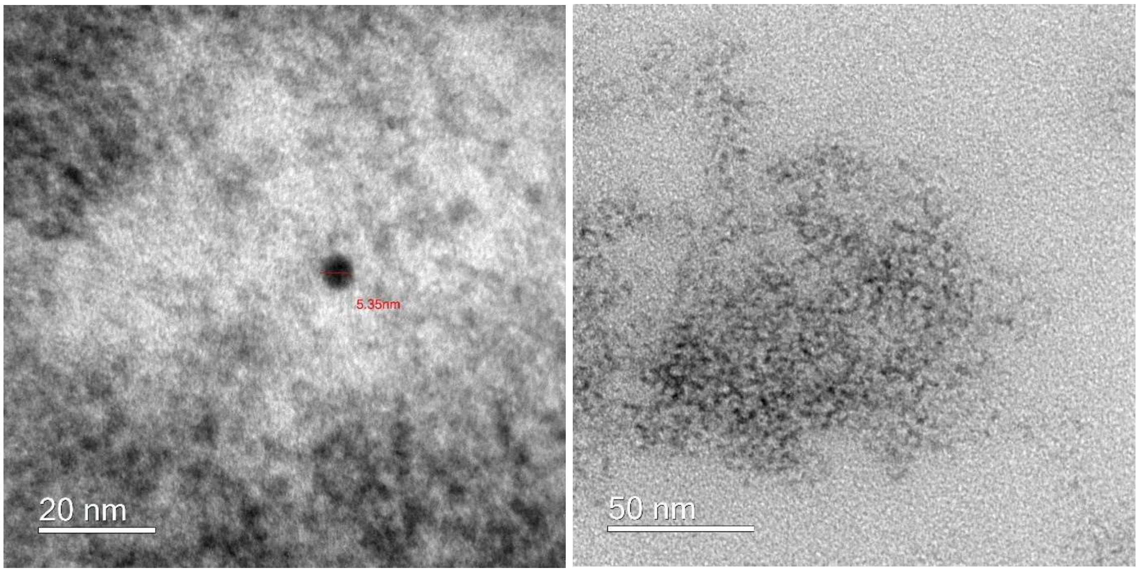

Figure 2): TEM images of CuInS2 (CIS) quantum dots.

3. EDX Characterisation of CIS-PDMS Fibre-Optic Films

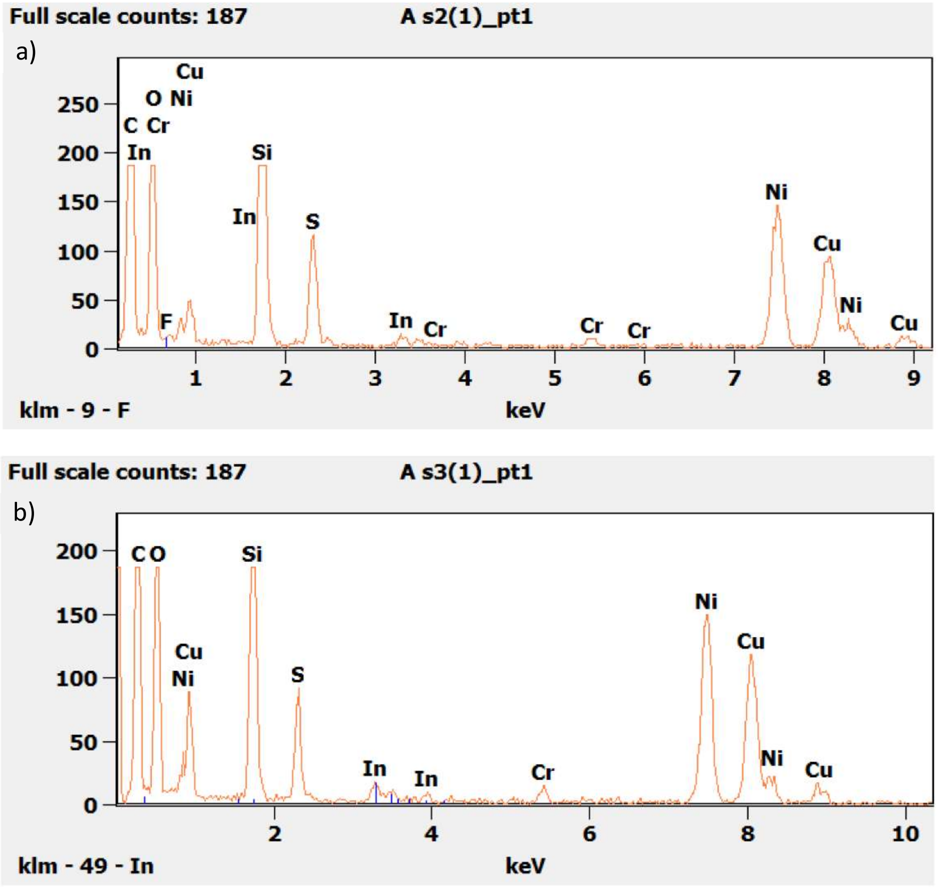

Figure 3): Energy Dispersive X-Ray Spectra of the two samples taken from the side (a) and distal end (b) of the CIS-PDMS-coated fibre.

4. Gel-Wax Ink-Filled Phantom Diagram

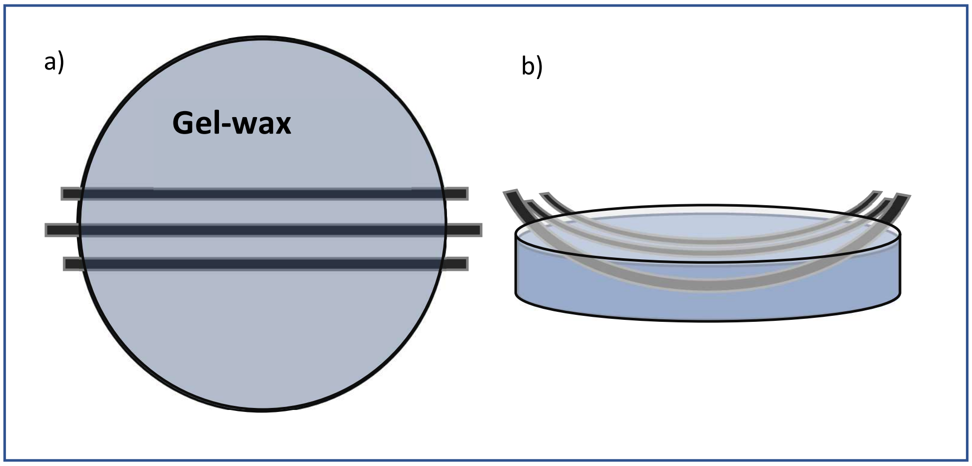

Figure 4): Schematic of ink-filled tubed phantom depicting both the above and side-on views.

5. Dual-Modality OpUS/PA Imaging Diagram

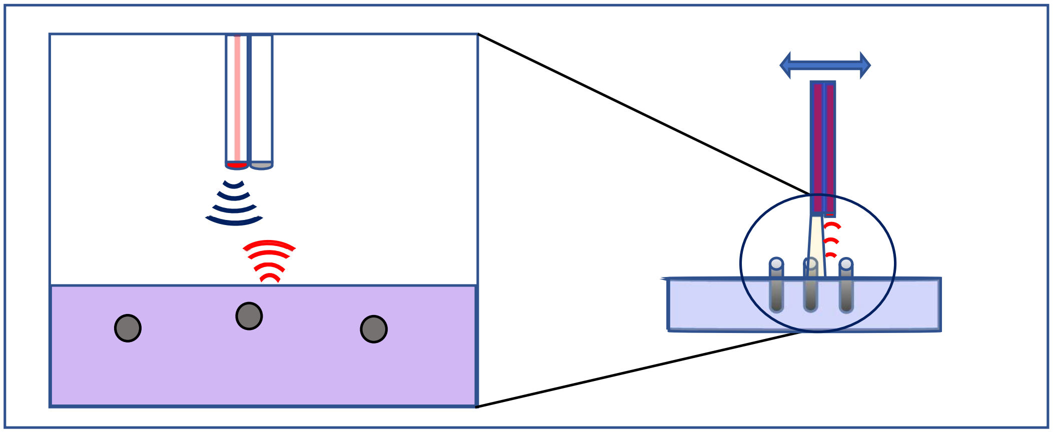

Figure 5): A schematic of fibre-optic OpUS transducer, formed from pairing CIS-PDMS coating with fibre-optic Fabry-Perot sensor, raster scanning an ink-filled tubed phantom depicting both the OpUS and PA imaging modes including a magnified view of the former.

6. OpUS, PA, and Overlaid OpUS/PA Images

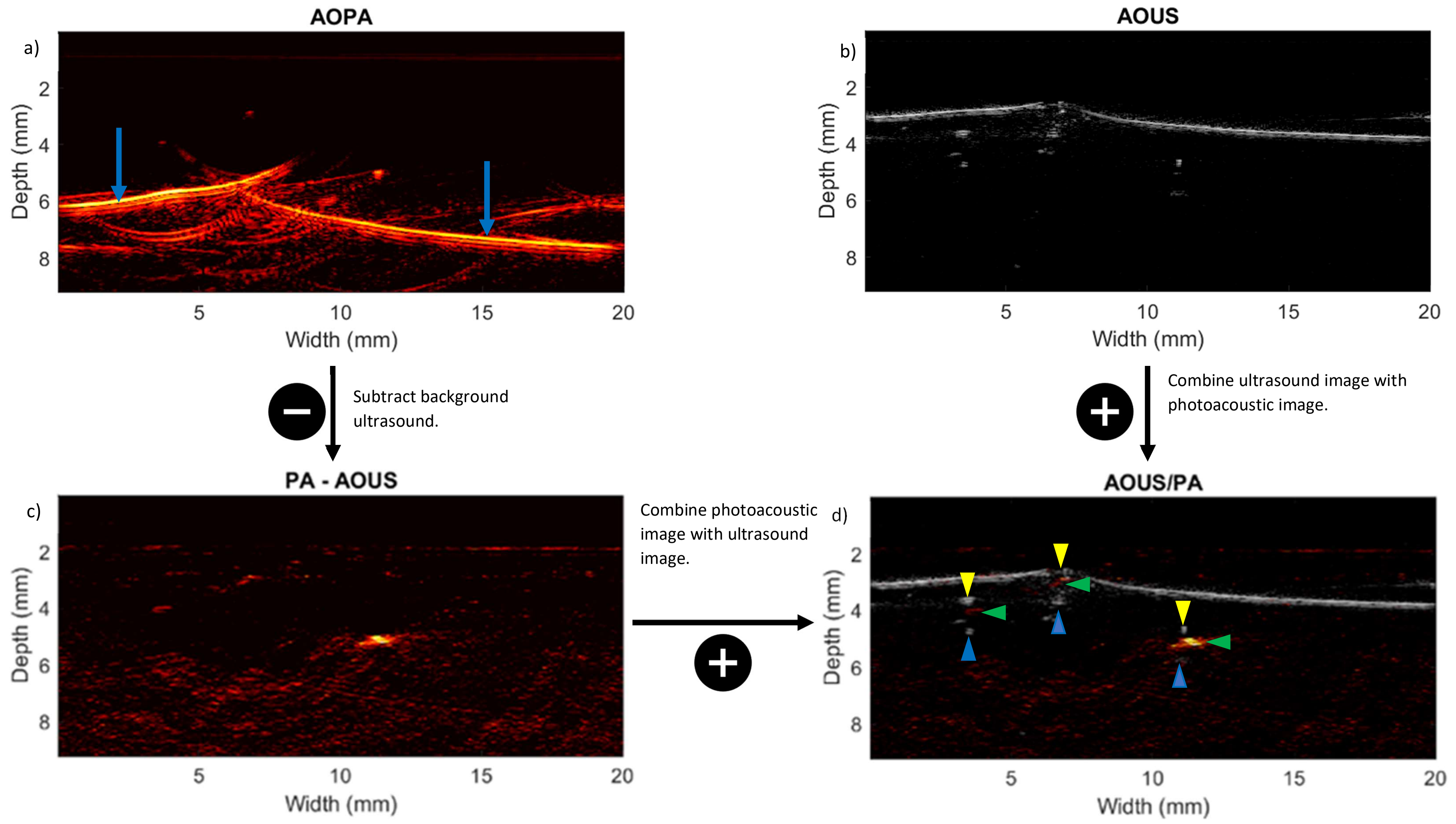

Figure 6): Acquired images of an ink-filled gel-wax phantom. a) Photoacoustic image with residual ultrasound signal highlighted by blue arrows. b) corresponding pulse-echo ultrasound image. c) Photoacoustic image from a) with ultrasound signal subtracted using a general linear model approach. d) Combined pulse-echo ultrasound (a)) and photoacoustic (c)) image. Arrows indicate locations of signals arising from the tubes. US top tube boundary – yellow arrow, US bottom tube boundary – blue arrow, PA signal – green arrow.

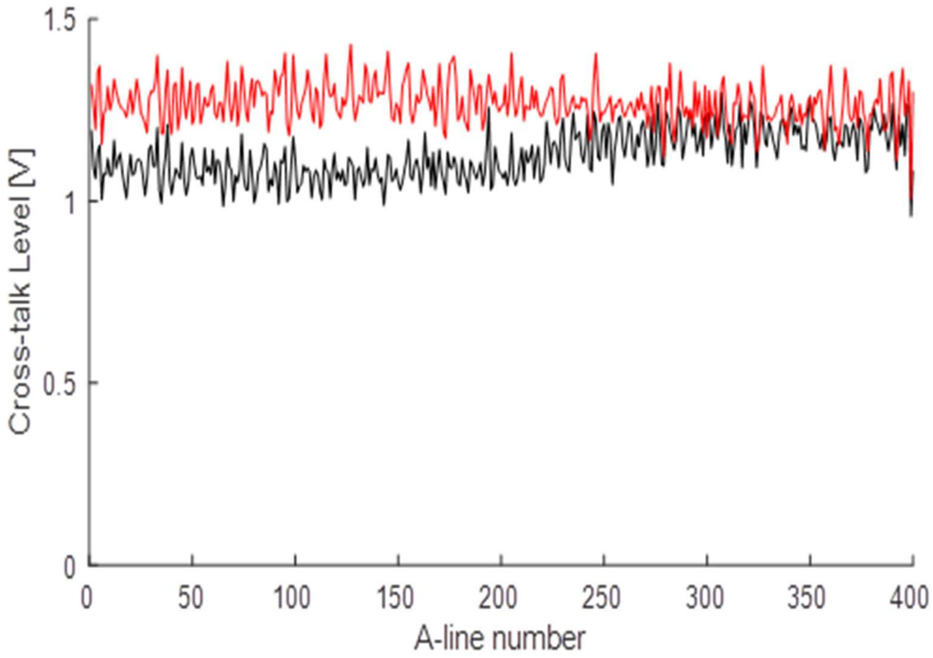

Figure 7): Peak positive (black) and negative (red) signal level recorded by the Fabry-Pérot sensor originating from ultrasound crosstalk, i.e. signal transmitted directly from the ultrasound generator to the Fabry-Pérot sensor, as a function of the time characterised by A-line scans over an extended period. Acquired at a rate of *ca.* 3 A-lines per second.

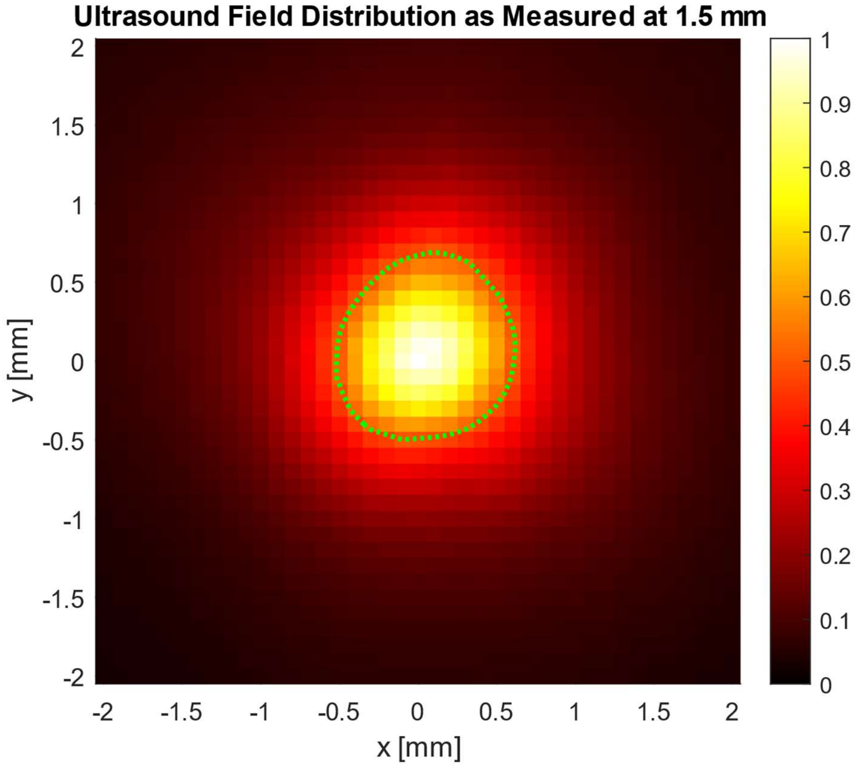

Figure 8): An example ultrasound field scan as measured at 1.5 mm from the CIS-PDMS coating. The field has been normalised to a maximum value of 1. The full-width half-maximum is shown by the dashed green contour line.
